# Genome-wide map of regulatory interactions in the human genome

**DOI:** 10.1101/gr.176586.114

**Published:** 2014-12

**Authors:** Nastaran Heidari, Douglas H. Phanstiel, Chao He, Fabian Grubert, Fereshteh Jahanbani, Maya Kasowski, Michael Q. Zhang, Michael P. Snyder

**Affiliations:** 1 Department of Genetics, Stanford University School of Medicine, Stanford, California 94305, USA;; 2 MOE Key Laboratory of Bioinformatics and Bioinformatics Division, Center for Synthetic and System Biology, TNLIST/Department of Automation, Tsinghua University, Beijing 100084, China;; 3 Department of Molecular, Cellular, and Developmental Biology, Yale University, New Haven, Connecticut 06520, USA;; 4 Department of Molecular and Cell Biology, Center for Systems Biology, The University of Texas at Dallas, Richardson, Texas 75080-3021, USA

## Abstract

Increasing evidence suggests that interactions between regulatory genomic elements play an important role in regulating gene expression. We generated a genome-wide interaction map of regulatory elements in human cells (ENCODE tier 1 cells, K562, GM12878) using Chromatin Interaction Analysis by Paired-End Tag sequencing (ChIA-PET) experiments targeting six broadly distributed factors. Bound regions covered 80% of DNase I hypersensitive sites including 99.7% of TSS and 98% of enhancers. Correlating this map with ChIP-seq and RNA-seq data sets revealed cohesin, CTCF, and ZNF143 as key components of three-dimensional chromatin structure and revealed how the distal chromatin state affects gene transcription. Comparison of interactions between cell types revealed that enhancer–promoter interactions were highly cell-type-specific. Construction and comparison of distal and proximal regulatory networks revealed stark differences in structure and biological function. Proximal binding events are enriched at genes with housekeeping functions, while distal binding events interact with genes involved in dynamic biological processes including response to stimulus. This study reveals new mechanistic and functional insights into regulatory region organization in the nucleus.

Since the sequencing of the human genome in 2001, great progress has been made in mapping the location of genes and other regulatory genomic elements. Systematic mapping of transcription factor binding sites and open chromatin regions have uncovered complex regulatory networks revealing mechanisms of gene regulation (The ENCODE Project Consortium 2012). However, increasing evidence suggests that in addition to local interactions, 3D contacts between distal regulatory elements play an important role in gene regulation (Gondor and Ohlsson 2009; Schoenfelder et al. 2010). Such contacts have been implicated in diverse biological phenomena including development, cancer, and immune response (Jhunjhunwala et al. 2008; Kenter et al. 2012; Rickman et al. 2012; Apostolou et al. 2013; Liu and Cheung 2013; Phillips-Cremins et al. 2013). However, due to the limited ability to study three-dimensional (3D) chromatin structure with sufficient breadth and resolution, our knowledge regarding the characteristics and impact of chromatin structure remains limited.

Numerous genomic technologies have evolved to interrogate 3D interactions, each with its own benefits and disadvantages. Virtually all of the experimental methods use cross-linking of chromatin, cleavage of DNA, and proximity ligation of interacting segments to create chimeric fragments of DNA. In the first iteration of this method, chromatin conformation capture (3C), these chimeric fragments were detected via PCR and electrophoresis (Dekker et al. 2002). In recent years significant advances in sequencing technology have spawned a variety of high-throughput variations (Dostie et al. 2006; Simonis et al. 2006; Zhao et al. 2006; Fullwood et al. 2009; Lieberman-Aiden et al. 2009). One such technology, Hi-C, allows genome-wide detection of interaction frequencies, typically at a resolution of 20–50 kb, and has been used to reveal megabase scale topologically associated domains (TADs) that organize human and mouse genomes. (Dixon et al. 2012; Gibcus and Dekker 2013). High-resolution detection of interacting fragments < 10 kb via Hi-C requires extremely deep sequencing but has recently been achieved and used to characterize chromatin interactions in human fibroblast cells (Jin et al. 2013). More commonly, high-resolution detection of interactions is achieved by targeting specific genomic regions with alternative techniques. For example, 5C technology allows high-resolution detection of interaction frequencies but is only currently feasible for small portions (∼1%) of the genome (Sanyal et al. 2012). Application of 5C to selected regions of the mouse genome revealed substantial changes in subTAD architecture between different cell types (Phillips-Cremins et al. 2013). However, the limited genomic coverage precludes the generation and characterization of genome-wide distal regulatory networks. A third technique, Chromatin Interaction Analysis by Paired-End Tag sequencing (ChIA-PET), uses a chromatin immunoprecipitation step that allows the detection of long-range interactions at regions bound by a target protein of interest. Enriching for loci bound by a specific protein drastically reduces the sequencing depth required to accurately detect interactions and enables detection of interactions between regions of < 5 kb. This technique has been utilized to study interactions involving subsets of functional genomic elements bound by estrogen receptor 1(ESR1), POLR2A, CTCF, SMC1A, and H3K4me2 (Fullwood et al. 2009; Handoko et al. 2011; Chepelev et al. 2012; Li et al. 2012; DeMare et al. 2013; Zhang et al. 2013).

Despite these advances, many questions regarding the nature of 3D chromatin interactions remain elusive: What factors and combinations of factors are involved in establishing and maintaining long-distance interactions? How do the chromatin states of distal interacting loci effect gene expression? How are these interactions used to regulate different biological functions? Answering these questions requires both a comprehensive map of interactions between regulatory elements and detailed maps of transcription factor (TF) binding, histone modification, and gene expression in the investigated cells.

To address these questions, we conducted ChIA-PET experiments targeting six chromosomal proteins/modifications broadly associated with transcriptional regulation in a well-characterized myelogenous leukemia human cell line (K562). The interrogated regions cover the vast majority of open chromatin regions and provide a genome-wide map of long-range interactions between regulatory elements. Through extensive integration with hundreds of available genomic data sets, we characterized the proteins involved in 3D chromosomal organization, the functional impact of interactions, and the general characteristics of distal regulatory networks. To address the function and specificity of these interactions, we also conducted ChIA-PET experiments targeting RAD21 in a well-characterized human lymphoblastoid cell line (LCL) and compared it with RAD21 anchored interactions in K562 cells.

## Results

### Genome-wide map of regulatory interactions

In order to detect regulatory interactions on a global scale, we conducted ChIA-PET in K562 cells using antibodies targeting six broadly distributed DNA binding proteins and histone marks (hereinafter referred to simply as “factors”). The factors include marks of enhancers, promoters, and active regulatory elements (H3K4me1, H3K4me2, H3K4me3, H3K27ac) as well as POLR2A and a component of the cohesin complex (RAD21). At least two biological replicate experiments were performed and interactions were scored and filtered using a novel ChIA-PET processing method (Supplemental Methods). This method determines both binding sites and interaction frequencies between any two bound loci. Due to a physical connection through the DNA molecule itself, random interactions occur at a rate that correlates with genomic distance (Dekker et al. 2002). To account for the influence of genomic distance on interaction frequency, we devised a resampling method that generates a control rewired data set that retains the same distribution of PET distances as the observed data set. For each pair of binding peaks, we calculate a *Z*-score by comparing the interaction frequency to the local mean and standard deviation of interaction frequencies from the rewired data set. *Z*-scores are calculated for both observed and rewired data sets, which allows interactions to be filtered to a user-defined false discovery rate (FDR). At an FDR of 10% we identified a total of 29,366 confident long-range interactions from the six factors/proteins (Supplemental Table S1). The median size of the interacting loci was 3603 bp, while the median distance between two interacting loci was 120,367 bp (Supplemental Fig. S1). Specific information for each data set and each factor can be found in Supplemental Tables S2 and S3.

**Figure 1.**
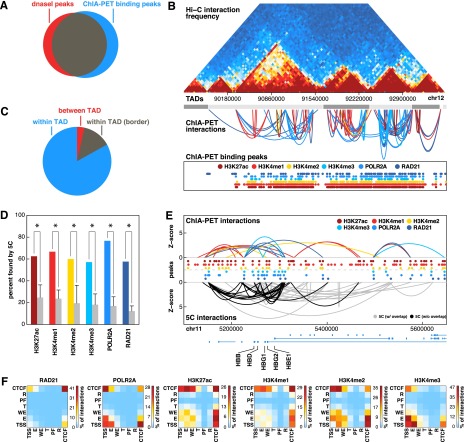
Comparison of ChIA-PET to Hi-C and 5C. (*A*) Venn diagram depicting overlap between binding peaks detected from ChIA-PET data sets (all six factors combined) and DNase I hypersensitive sites. A total of 80% of DNase I peaks are bound by at least one ChIA-PET peak. (*B*) Comparison of interactions found by ChIA-PET and Hi-C data sets. Hi-C interaction frequencies for human embryonic stem cells are shown in the *top* panel. TADs determined by Hi-C are represented by alternating light and dark gray boxes. ChIA-PET interactions are represented by arches. The height of each arch is proportional to the *Z*-score of each interaction and the color indicates in which data set it was detected. ChIA-PET binding peaks are shown at the *bottom*. (*C*) Pie chart depicting the number of ChIA-PET interactions that are completely within one TAD (blue), within one TAD and overlap a TAD border (gray), and between two TADs (red.) TAD borders were defined by extending borders 20 kb in both directions. (*D*) Percentage of ChIA-PET interactions also found by 5C. Only ChIA-PET interactions that were tested by 5C were considered. Gray bars represent expected percentages generated by randomly selecting interactions from the tested 5C region while retaining the same distribution of interaction distances. (*) *P*-value < 0.05 (permutation testing, 1000 permutations). (*E*) ChIA-PET interactions (*top*) and 5C interactions (*bottom*) at the globin locus on chromosome 11. Heights of arches represent *Z*-scores of interactions. ChIA-PET interactions are colored according to data sets. 5C interactions are colored according to whether or not they overlap a ChIA-PET interaction (black: yes, gray: no). Binding peaks for each data set are represented by colored circles. (*F*) Heat maps depicting the percentage of interactions from each data set that connect different types of genomic loci.

To better understand the nature of each interaction, we compared our ChIA-PET results to the extensive list of elements defined by the ENCODE Project Consortium (2012). We first defined a set of “regulatory elements” in K562 cells by intersecting chromatin state calls as determined by Hoffman et al. (2013) and DNase I hypersensitive sites (DHSs) as defined by Thurman et al. (2012). Each DHS was assigned to a single chromatin state based in the chromatin state it overlapped, with the most resulting in 169,871 annotated regulatory elements and 32,395 undefined elements that did not overlap any chromatin state calls (Table 1; see Supplemental Methods for details). The binding peaks detected from our six ChIA-PET data sets covered the majority of DNase I hypersensitive sites (80%) and annotated regulatory elements, (82.7%) including 99.7% of TSS and 98.0% of enhancers (Fig. 1A; Table 1). A total of 44% of CTCF regions, 36% of promoter regions, and 21% of enhancers were involved in at least a single interaction (Table 1; Supplemental Table S4).

**Table 1.**
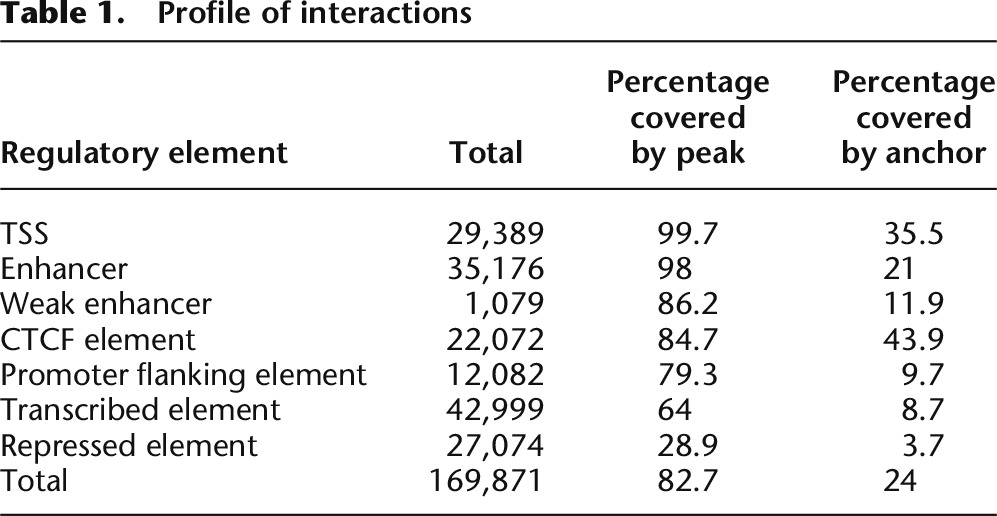
Profile of interactions

ChIA-PET interactions are highly consistent with interactions determined by both Hi-C and 5C (Fig. 1). Genome-wide interactions maps generated by Hi-C suggested that genomes are organized in megabase scale topologically associated domains (TADs) characterized by high intradomain interaction frequencies. Consistent with these findings, the vast majority of our ChIA-PET interactions (97%) connected two regions within the same topological domain (Fig. 1B,C).

High-resolution interaction maps have been generated for K562 using 5C technology, albeit for only a small fraction of the genome (∼1%). Within these regions 62.5% of ChIA-PET interactions were also identified by 5C. This overlap is significantly higher than expected (19.2%) based on permutation testing (*P* < 0.001) (Fig. 1D). Comparison to previous ChIA-PET data sets targeting POLR2A in K562 cells reveals that a greater fraction of our interactions agree with 5C results (Supplemental Fig. S1A; Li et al. 2012). This improvement in accuracy of interaction calling can be attributed to increases in sequencing depth as well as the distance-dependent scoring method and stringent empirical FDR filtering we used.

Comparison of the types of interactions that comprised each data set revealed the similarities and differences between each TF (Fig. 1F). For example, 41% of RAD21-bound interactions linked two CTCF elements and only 7% connected enhancers to promoters. In contrast, 47% of H3K4me3-bound interactions connected enhancers to promoters but only 28% connected two CTCF elements. Despite these differences the majority of interactions were detected in more than one data set. A total of 19,007 (65%) of interactions were found in more than one data set while 10,359 (35%) were factor-specific. The total numbers of general and factor-specific interactions for each data set are detailed in Supplemental Table S3 and displayed in Supplemental Figure S1D.

Given the agreement between previous interaction studies and the comprehensiveness of the coverage we conclude that our data set represents a genome-wide map of long-range interactions between regulatory elements in K562 cells.

### Cohesin, CTCF, ZNF143, and HOT regions are enriched at interacting loci

**Figure 2.**
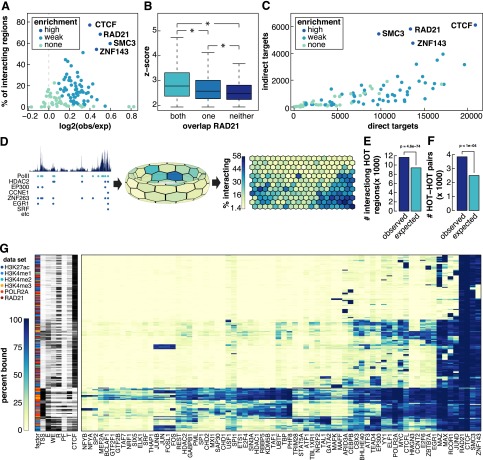
Factors enriched at interacting loci. (*A*) TF enrichment at interacting loci. The *x*-axis represents the log_2_ ratio of observed divided by expected TF binding peaks that overlap interacting loci. The *y*-axis represents the number of interacting loci at which that factor is bound. Colors of circles represent the level of enrichment (see Supplemental Methods). (*B*) Box and whisker plot of *Z*-scores of interactions that overlap a RAD21 peak at both, one, or neither end of an interaction. (*) Significant differences (*P* < 0.05, Wilcoxon signed-rank test). (*C*) Scatter plot comparing the number of direct and indirect targets of each TF. Colors correspond to how enriched that factor was at interacting loci. (*D*) Depiction of how SOM maps were generated using POLR2A as an example. (*Left*) Binding profile of POLR2A in K562 cells as well as POLR2A peaks (light blue circles) and peaks of other TFs that overlap POLR2A peaks (dark blue). (*Middle*) Toroidal depiction of SOM generated from POLR2A data set. Each hexagon represents a neuron comprised of POLR2A binding peaks that share patterns of TF cobinding. (*Right*) Planar view of POLR2A SOM map. Neurons are colored to depict the percentage of POLR2A peaks in each neuron that are involved in an interaction. (*E*) Barplot showing number of observed and expected HOT regions involved in interactions. *P*-values were determined by Fisher’s exact test. (*F*) Barplot showing number of observed and expected number of interactions linking two HOT regions. Significance was determined by permutation testing. (*G*) Heatmap representing neurons that are significantly enriched for interactions (*P* < 0.01, Fisher’s exact test with Benjamini-Hochberg correction, fold-enrichment > 2). For visualization purposes, only selected TFs are shown. The data sets in which a neuron was detected are shown on the far *left* as well as the percentage of peaks in each neuron that overlapped different types of annotated DHSs.

To determine which TFs participate in long-range interactions, we intersected our data with binding regions of 102 TFs determined via ChIP-seq by the ENCODE Project Consortium (2012). Four factors were strongly enriched at interacting loci; RAD21, SMC3, CTCF, and ZNF143 (Fig. 2A; Supplemental Fig. S2A). RAD21 and SMC3 are members of the cohesin complex, a complex traditionally known for tethering together sister chromatids during mitosis. However, cohesin complex is bound to DNA throughout most of the cell cycle and has been recently implicated in long-range interactions (Kagey et al. 2010; Hoffman et al. 2013; Merkenschlager and Odom 2013; Phillips-Cremins et al. 2013). CTCF, a canonical insulator protein, which is found at the majority (96.7%) of RAD21 sites in K562 cells, has also recently been implicated in long-range interactions (Handoko et al. 2011). ZNF143 is a zinc finger protein known as a transcriptional activator, which often colocalizes with CTCF and cohesin (Xie et al. 2013). ZNF143 has been shown to regulate both coding and noncoding genes by binding an 18-bp motif located on the core promoter region, but to date has not been implicated in the regulation of 3D chromatin structure (Schaub et al. 1997; Ngondo-Mbongo et al. 2013). These four factors were enriched both when the experimental data were pooled (Fig. 2A) and when each factor was analyzed separately (Supplemental Fig. S2A).

We next determined whether the strength of an interaction correlated with the presence or absence of RAD21. Indeed, for all data sets interactions with RAD21 bound at both ends had significantly higher *Z*-scores relative to interactions where RAD21 was not bound at either end or bound at only one end (*P* < 0.05, Wilcoxon signed rank test) (Fig. 2B; Supplemental Fig. S2B). Finally, we compared the number of proximal targets (TF binds at gene promoter) and distal targets (TF binds at distal loci that interacts with gene promoter) of each TF. While most TFs regulate target genes via both proximal and distal binding, RAD21, SMC3, CTCF, and ZNF143 had the highest ratios of distal vs. proximal targets (Fig. 2C). Taken together these results implicate cohesin, CTCF, and ZNF143 as the central regulators of long-range interactions. These findings also suggest that these factors act at both ends of an interaction.

We next determined whether certain combinations of factors were enriched at interacting genomic loci (Fig. 2D–G). For each TF we built a self-organizing map (SOM) which clusters genomic loci into neurons based on the combinations of factors that are bound (Fig. 2D). Multiple neurons were enriched for interactions indicating that certain TF coassociations do correlate with long-range interactions. To elucidate which combinations of TFs were enriched, we constructed a heatmap displaying the combinations of TFs in each neuron (Supplemental Fig. 2G). Note, only neurons that were significantly enriched (*P* ≤ 0.01, Fisher’s exact test with Bonferroni correction) for interactions are shown. RAD21 and CTCF and SMC3 were present at very high percentages in virtually all of the neurons that were enriched for interactions. ZNF143 were in most but not all such neurons. These results underscore the central role of these four factors in establishment or maintenance of (3D) chromatin structure. Several other small clusters are evident including neurons with high percentages of enhancer-related TFs such as EP300 and TEAD4. Another cluster of neurons enriched in interactions is characterized by the presence of AP-1 family (FOS and JUN) proteins which are known as early response transcription factors and are involved in cell proliferation, differentiation, and survival as well as other important cellular events (Ameyar et al. 2003).

Many of the neurons in this plot were characterized by cobinding of many TFs (Fig. 2E,F). These neurons tended to contain TSS regions and may represent high-occupancy target (HOT) regions, genomic loci bound by multiple TFs that often mark the promoters of highly expressed genes (Moorman et al. 2006; Gerstein et al. 2010; Negre et al. 2011; Yip et al. 2012). We found that HOT regions, as determined by Boyle and colleagues were significantly enriched in long-range interactions (*P* = 5.9 × 10^−62^, Fisher’s exact test) and that HOT regions had a strong preference to interact with other HOT regions (*P* = 4.5 × 10^−271^, Fisher’s exact test) (Boyle et al. 2014). Taken together these findings support the model that DNA looping brings active promoters into distinct nuclear subcompartments termed “transcription factories.”

### Functional impact of interactions between promoters and distal regulatory elements

Physical interaction of gene promoters with distal regulatory elements represents an important mechanism of gene regulation. Understanding these interactions requires both mapping the interactions between promoters and distal regulatory elements and assessing their functional significance. Our genome-wide map of regulatory interactions intersected with a broad range of other data sets acquired on K562 cells and other well-characterized cell lines allows us to achieve a detailed understanding of the functional impact of long-range interactions at a level not previously achieved.

**Figure 3.**
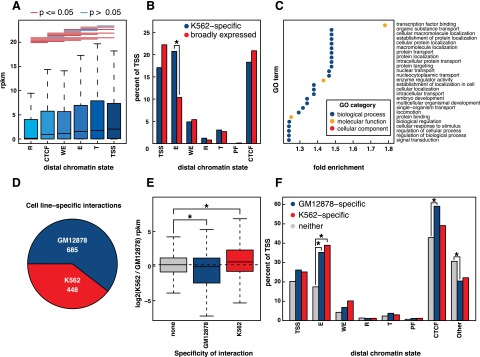
Promoter interactions. (*A*) Box and whisker plot depicting gene expression (RPKM) as a function of distal chromatin regions. Comparisons that exhibited significant differences (*P* < 0.05, Wilcoxon signed-rank test) are indicated by red lines, while those that were not significantly different (*P* ≥ 0.05, Wilcoxon signed-rank test) are indicated by blue lines. (*B*) Barplot depicting the percentage of genes whose promotors are involved in an interaction with each type of distal chromatin region. Values are shown for both genes that are K562-specific and genes that are broadly expressed. (*) Significant difference (*P* < 0.05, Fisher’s exact test). (*C*) GO terms enriched in sets of genes whose promoters interact with enhancers (*P* < 0.01, Fisher’s exact test with Benjamini-Hochberg correction, fold-change > 1.2). (*D*) Differential interactions between GM12878 and K562 (FDR = 0.05). See Supplemental Information. (*E*) Log_2_ expression changes between K562 and GM12878 for all genes, genes that overlap the ends of GM12878-specific interactions, and genes that overlap the ends of K562-specific interactions. (*) Significant difference from all genes (*P* < 0.01, Wilcoxon signed-rank test). (*F*) Percentage of TSS that interact with at least one of each distal regulatory region. (*) Significant difference from all genes (*P* < 0.01, Fisher’s exact test).

We first investigated the effect that chromatin state of a distal interacting region had on gene expression (Fig. 3A). We observed a clear trend in which interactions with transcription start sites (TSS), transcribed regions (T), or enhancers (E) resulted in high gene expression, interactions with weak enhancers (WE) or CTCF regions resulted in moderate gene expression, and interactions with repressed (R) regions resulted in low gene expression. These results have several important implications. First, these results clearly indicate that (3D) chromatin structure does significantly correlate with transcriptional activity and is therefore an important characteristic to consider when building regulatory networks and models of regulation. Second, these findings suggest that all types of distal chromatin states have an impact on gene expression and that the type of distal chromatin state is a predictor of the functional impact.

Previous studies have shown that enhancers are highly cell-type-specific compared to other types of regulatory elements (Heintzman et al. 2009). Since our data maps all types of regulatory elements to their targets we sought to determine whether interactions with any types of regulatory elements correlated with cell-type-specific expression of the genes with which they interacted. Genes that were expressed specifically in K562 cells compared to 11 other studied cell lines (see Methods) were enriched more than twofold for K562 enhancer–promoter interactions (*P* < 0.05, Fisher’s exact test) (Fig. 3B). Interestingly, no other types of interaction exhibited this enrichment. Although not statistically significant, promoter–promoter interactions were enriched in broadly (i.e., constitutively) expressed genes.

Given the functional importance of enhancers in regulating transcription and establishing cell-type-specific gene expression patterns we next determined which Gene Ontology (GO) terms were enriched in genes whose promoters interacted with enhancers. Interestingly, genes corresponding to “transcription factor activity” were highly enriched (1.7-fold, *P* = 0.00079, Fisher’s exact test with Benjamini-Hochberg correction). This suggests an expanded role for enhancer–promoter interactions in regulatory networks as they may regulate not only these TFs but also the downstream targets of these TFs. We investigate the role of distal interactions in regulatory networks in greater detail later in this work (vide infra). Many of the TFs whose promoters are involved in interactions with enhancers regulate blood and immune-related functions including hematopoietically expressed homeobox protein (HHEX), nuclear factor of kappa light polypeptide gene enhancer in B-cells inhibitor, alpha (NFKBIA), and interferon, gamma-inducible protein 16 (IFI16) (Trapani et al. 1992; Grilli et al. 1993; Liao et al. 2000).

### Cell-type-specific interactions are enriched for enhancer-promoter interactions and correlate with differences in gene expression

In order to determine how interactions differed between cell types, we conducted ChIA-PET experiments targeting RAD21 in the well-characterized human LCL GM12878. We then determined differential interactions between the two cell lines while controlling for differences in RAD21 binding (see Supplemental Information). At an FDR of 0.05 we found 1133 differential interactions, 685 and 448 interactions specific to either GM12878 or K562, respectively (Fig. 3D). Genes whose promoters overlapped the ends of a cell line-specific interaction were expressed at significantly higher levels in that cell type (Fig. 3E) (*P* < 0.01, Wilcoxon signed rank test), indicating that these interactions likely play a regulatory role and are specific to certain cell types. We next compared the percentage of TSS that interacted with each type of distal regulatory element. As shown in Figure 3F, TSS-enhancer interactions were highly enriched in the cell line-specific interactions (*P* < 0.01, Fisher’s exact test). While enhancers have been known to be the most variable regulatory type, this is the first time a global genomic analysis has demonstrated that enhancer–promoter interactions are more variable than any other type of promoter centered interaction.

### Loop regions correspond to functional chromatin domains

**Figure 4.**
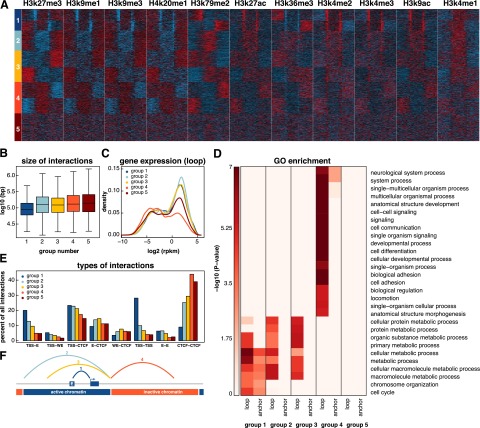
Chromatin domains revealed by clustering interactions. (*A*) Interactions were clustered into five categories based on the distribution of 11 histone marks. Normalized signals for 11 histone marks were determined in 30 equally sized bins (10 on either side of the interaction and 10 within the loop of the interaction). Interactions were then clustered in eight groups using k-means clustering. Symmetrical groups were grouped to give rise to five final groups. (*B*) Box and whisker plot depicting the size distribution of interactions in each of the five groups. (*C*) Density plot showing gene expression values of genes whose promoters reside in the loop regions of each group. (*D*) GO biological processes enriched in the anchor and loop regions of each of the five groups (*P* < 10^−5^, Fisher’s exact test with Benjamini-Hochberg correction). (*E*) Barplot depicting the percentage of interactions in each of the five groups that links certain types of annotated regulatory elements. (*F*) Schematic diagram of the loops in each of the first four groups.

Another mechanism of transcriptional regulation may involve the compartmentalization of DNA into large domains of active or inactive chromatin (i.e., TADs and subTADs). It has been previously shown that boundaries of megabase scale topological domains demarcate transitions from active to inactive chromatin and that CTCF is enriched at these boundaries (Dixon et al. 2012). In addition, CTCF-bound interactions marked borders of active and inactive chromatin in mouse embryonic stem cells (Handoko et al. 2011). We reasoned that long-range interactions might form loops affecting gene expression not only of the genes at the anchor regions but also within the loop region itself. To investigate this, we clustered interactions by histone mark occupancy both within and adjacent to each loop, as described by Handoko et al. (2011) (Fig. 4A). Five distinct groups emerged.

Group 1 is characterized by short interactions, active marks in the anchor regions, and enrichment for TSS–TSS and E–TSS interaction types (Fig. 4A–E). This group likely represents regulatory looping events that bring together promoters and distal regulatory elements. Indeed, anchor regions of group 1 are enriched for a number of GO terms (e.g., cell cycle and metabolic process). In contrast, groups 2, 3, and 4 showed no enrichment for histone marks or GO terms at the anchor regions (Fig. 4A,D). Instead, they were characterized by relatively long interaction distances, GO enrichment inside the loop region, and coordinated gene expression (Fig. 4B–D). Loops in groups 2 and 3 harbored active histone marks, exhibited high gene expression, and were enriched in GO terms including “metabolic processes” and “chromatin organization” (Fig. 4C,D). Genes within the loops of group 4, which harbored inactive regions, were enriched in a completely different set of GO terms including “signaling,” “developmental process,” and “cell adhesion” (Fig. 4A,D). Group 5, which has no characteristic histone pattern, is characterized by long interactions that show no GO enrichment either at the anchors or within the loops.

These analyses allowed us to create models for each group of interactions (Fig. 4F). Group 1 interactions bring together promoters with other promoters or other regulatory elements to affect transcription in the anchor regions. Group 2 interactions harbor active regions of chromatin. Group 3 interactions reach from the border of an active region into the middle of an active region. These interactions may provide structural support for larger active loops. Group 4 interactions reach from borders into inactive regions of chromatin. The role of group 5 interactions is unclear.

In total, these findings suggest that long-range chromatin interactions may function in at least two ways to regulate gene expression: (1) by bringing together promoters and distal regulatory elements and (2) by creating large structural domains that harbor functionally related genes that share gene expression patterns.

### Distinct architecture of proximal and distal regulatory networks

Having established both the quality and functional relevance of these long-range interactions we next constructed and characterized the K562 distal regulatory network using the wealth of binding data in these cells. Comparison of the distal regulatory network to the proximal regulatory network revealed substantial differences in TF-target relationships, hierarchical structure, and network motif usage. Integration of the regulatory information from both networks into a single combined regulatory network provides a comprehensive view of regulatory TF binding in K562 cells.

**Figure 5.**
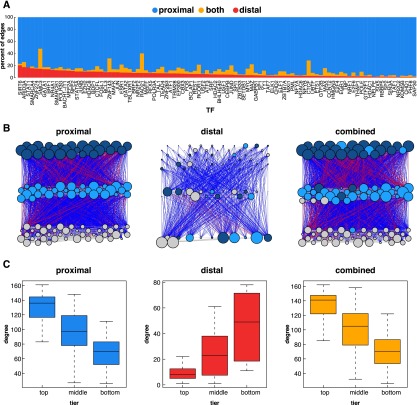
Proximal vs. distal network architecture in K562 cells. (*A*) For each TF, the percentage of targets found in the distal, proximal, or both networks is depicted. (*B*) Hierarchical networks built from proximal, distal, and combined TF-only networks. Blue lines represent downward edges, red lines represent upward edges, and gray lines represent lateral edges. The colors of the nodes represent the tier in which the node resides in the proximal network. The size of the node represents the degree (total number of inward and outward edges) for each node in that network. (*C*) Box and whisker plots depicting the degree (total inward and outward edges) of nodes in each tier of each hierarchical network.

We first compared proximal and distal targets for each of the TFs for which ENCODE TF binding data was available (Fig. 5A). A total of 58% of the edges present in the distal network were not found in the proximal network indicating that overall these networks are highly dissimilar. Four factors, however, showed notably high redundancy between the proximal and distal networks: RAD21, SMC3, CTCF, and ZNF143 (Fig. 5A). This finding is consistent with the binding of these factors at both ends of an interaction and agrees with our earlier findings that cohesin’s presence at both ends of an interaction correlates with higher interaction frequency.

Next, TF networks were organized into three-tiered hierarchical networks as described by Gerstein and colleagues (Fig. 5B; Supplemental Fig. S3). A simulated annealing procedure was used to maximize downward-pointing edges (Gerstein et al. 2012). The color of the nodes corresponds to the tier of the node in the proximal network. Surprisingly, the tier assignments in the distal network show virtually no correlation with the proximal network (percent agreement: 36%). Other characteristics also distinguished the two hierarchical networks. For example, the proximal network has the fewest tier 1 TFs and the most tier 3 TFs, but that pattern is exactly opposite in the distal network (Supplemental Fig. S3D,E). In addition, the overall degree—the sum of all inward and outward edges—of the nodes in the proximal network decreased from top to bottom, but was increased in the distal regulatory network (Supplemental Fig. S3B). These trends were almost exactly the same in networks built for the GM12878 cell line, providing a further layer of confirmation (Supplemental Fig. S3). The combined network shows similar properties to the proximal network, which is expected since the proximal network contains more than twice as many edges. Nevertheless, the combined network likely provides the most accurate depiction of the regulatory infrastructure as it embodies both proximal and distal types of regulation.

### TFs regulate different classes of proteins via proximal and distal binding

**Figure 6.**
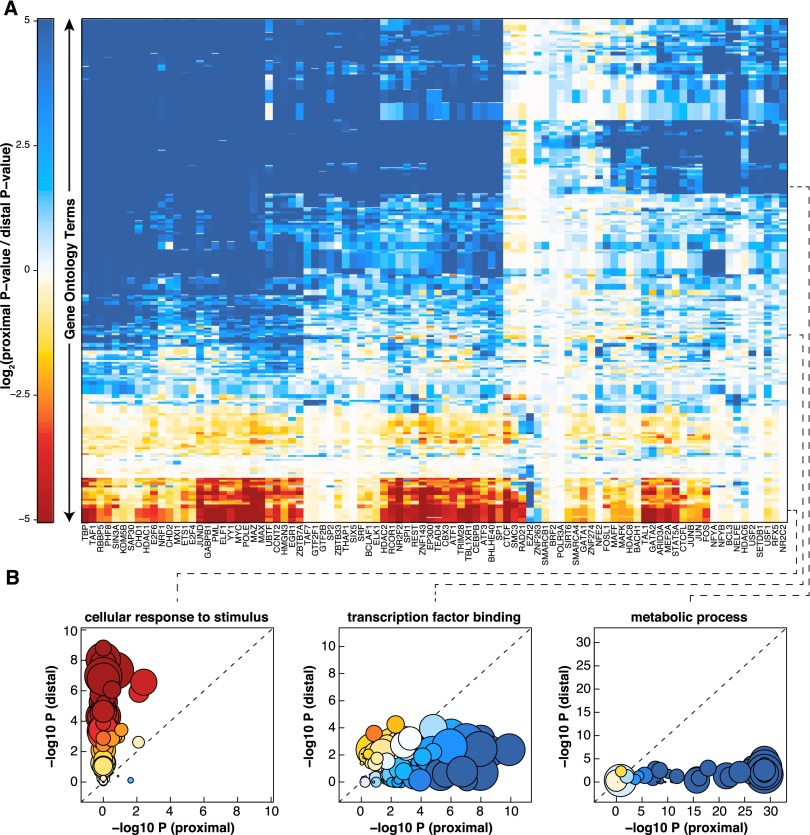
Proximal vs. distal regulation of GO terms. (*A*) Heatmap comparing enrichment of GO terms in proximal vs. distal targets of each TF. Each row corresponds to a GO term. Each column corresponds to a transcription factor. Red indicates greater enrichment in distal targets. Blue represents greater enrichment in proximal targets. (*B*) Three plots highlighting examples of GO terms that exhibit different profiles of enrichment. Each circle represents a TF. The size of the circle represents the number of targets in that GO term that the TF factor regulates (both proximally and distally). The color of the circle represents the relative enrichment (proximal vs. distal) using the same scale as shown in *A*.

Given the substantial differences in structure between the proximal and distal regulatory networks, we next asked whether there were differences in the biological processes that were regulated by each network. To do so we performed Gene Ontology enrichment analysis for the direct and indirect targets of each TF. We then calculated the log_2_ ratio of proximal vs. distal *P*-value and plotted the ratio as a heatmap (Fig. 6A). Positive values indicate stronger enrichment in proximal targets and negative values reflect stronger enrichment in distal targets. Many GO terms were mediated almost exclusively by proximal binding of TFs and tended to reflect general housekeeping functions of the cell including “cellular metabolic process,” “cell cycle,” and “mRNA processing.” In contrast, GO terms enriched via distal TF binding included more dynamic and cell-type-specific processes including “signal transduction,” “immune system process,” and “response to stimulus.” Other processes, such as “transcription factor binding,” were regulated both by proximal and distal binding events (Fig. 6A; Supplemental Fig. S4A). We performed the same analysis with networks built from GM12878 ChIP-seq and ChIA-PET data with similar results (Supplemental Fig. S5A). Most GO terms were regulated predominately by direct binding, though some GO terms were regulated by distal binding of certain TFs. In agreement with the K562 network, “metabolic process” was regulated almost exclusively by proximal binding and “transcription factor binding” was regulated by both distal and proximal binding events (Supplemental Fig. S5B). In contrast to the K562 network, “cellular response to stimulus” was regulated by both distal and proximal binding events (Supplemental Fig. S5B).

We next examined whether any TFs regulated different types of biological process via proximal and distal interactions (Supplemental Fig. S4B). While some TFs, including TAF7 and EZH2, only exhibited proximal enrichment for GO terms, others showed both proximal and distal enrichment. Interestingly, for many such TFs the processes regulated via proximal binding were completely different from those regulated via distal binding. For example, GATA2 regulates “biosynthetic process” and “cell cycle” via proximal binding but “cell differentiation” and “developmental process” via distal binding. Thus, at least some TFs likely mediate different biological processes via proximal vs. distal binding. CTCF and RAD21 tend to regulate the same biological processes via direct and indirect binding, which again highlights their occupancy at both ends of most detected interactions.

## Discussion

Combining multiple ChIA-PET data sets, we generated a genome-wide map of interactions between regulatory elements in human cells. Distance-dependent interaction scoring and filtering accurately identified long-range interactions, as confirmed by intersection with previous Hi-C and 5C studies. Analysis of these data sets provided novel insights into 3D chromatin structure, transcriptional regulation, and network wiring, and gives rise to a number of new models of gene regulation.

One of the most striking trends observed in this study regards the strong enrichment of CTCF, cohesin, and ZNF143 at interacting loci. These results agree with a study by Phillips-Cremins et al. (2013) who investigated interactions in select regions of the mouse genome and found that > 80% of interactions were anchored by some combination of CTCF, MED12, or SMC1, a subunit of cohesin. Our analysis further revealed that CTCF and RAD21 were members of nearly every cobinding pattern that was enriched for interactions. This differs from that observed in mouse by Phillips-Cremins et al. (2013), who found distinct sets of interactions occupied by CTCF without cohesin and cohesin without CTCF. These differences between the two studies may represent an important difference in chromatin structure between species or between different cell types or different sensitivities in the experimental methods. The role of ZNF143 as a transcriptional activator with binding at promoters of coding and noncoding genes is known. Recently, investigators introduced this factor as important in the maintenance of pluripotency of embryonic stem cells (Chen et al. 2008; Chia et al. 2010). Our findings implicate ZNF143 in long-range chromatin interactions for the first time. Further investigation is required to elucidate the specific role of this protein in the establishment and maintenance of the chromatin structure.

Comparing the results from these six data sets can help guide future experiments. While each data set revealed factor-specific interactions, the majority of interactions were found in more than one data set (Supplemental Fig. S1D). The RAD21 data set revealed more than twice as many interactions as any of the other data sets. Moreover, all data sets revealed strong enrichment of CTCF and cohesin proteins in their anchor regions.

HOT regions were enriched at interacting loci and tended to interact with other HOT regions. This finding supports the transcription factory model in which promoters of actively transcribed genes occupy distinct nuclear subcompartments. The strong enrichment for cohesin, CTCF, and ZNF143 at all interacting loci including HOT regions implicates these factors as possible regulators or facilitators of transcription factories.

We demonstrated that the chromatin state at distal regulatory regions correlates significantly with gene expression. Sanyal et al. (2012) had previously shown that genes whose promoters interact with enhancers are more likely to be expressed than genes whose promoters do not interact with enhancers. We extend those findings to show a gradient of expression values as a function of distal chromatin state in which TSS, T, and E are associated with high gene expression, WE and CTCF are associated with moderate gene expression, and R is associated with low gene expression. Further, we found that enhancer–promoter interactions, but not other types of interactions, tend to be cell-type-specific and are enriched at genes with the annotation “transcription factor binding.”

In addition to bringing together two functional elements, long-range interactions appear to form large loops that coincide with functionally coordinated domains of active and inactive chromatin. While this characteristic is shared with TADs, these loops tend to be substantially smaller and may represent subTAD structures. Further work is required to determine whether these looping events are a cause or result of chromatin boundaries; however, a recent study by Nora et al. (2012) has demonstrated that removal of boundary regions containing CTCF binding sites can result in loss of a TAD boundary.

Finally, we compared network wiring and architecture between proximal and distal regulatory networks and found substantial differences in TF-target relationships and network hierarchy that were consistent across cell lines. GO analysis revealed that many cell-type-specific and dynamic processes were regulated more by distal than proximal binding of TFs, while more routine biological processes tended to be regulated via proximal binding of TFs. We also show that certain TFs regulate distinct processes via proximal or distal binding.

During the preparation of this work, a related study appeared that mapped interactions at high resolution with Hi-C using very deep sequencing (Jin et al. 2013). Their study was performed in a different cell type, making direct comparisons difficult. One important distinction between the studies is that ours was carried out in one of the most well-characterized cell lines available. Extensive integration with hundreds of existing ChIP-seq and RNA-seq data sets allowed novel insights into the combination of factors involved in interactions, the effect of distal chromatin state on transcription, and the structure and function of distal regulatory networks. Such advances would not have been possible without both a comprehensive map of interacting regulatory regions and a compendious set of transcription factor and histone marks binding profiles. Generated in one of the most well-studied human cell lines, this study reveals not only many new insights but also serves as a valuable resource for the scientific community.

## Methods

### Cell culture and ChIA-PET library preparation

All ChIA-PET experiments were conducted for this study and are not previously published. K562 (ATCC# CCL-243) cells were grown under standard culture conditions and harvested at log phase. The cells were cross-linked by 1.5 mM EGS for 20 min followed by adding 1% formaldehyde at room temperature for 10 min on a plate rotator and then neutralized with 0.2 M glycine. After a two-step of cell lysis and nuclear lysis with RIPA buffer, chromatin was sheared by BioRuptor 300 to an average size of 500 bp. Sheared chromatin was subjected to overnight Immunoprecipitation by the addition of 50 mg of antibodies. The antibodies used in this study were POLR2A monoclonal antibody 8WG16 (Covance, MMS-126R), rabbit polyclonal H3K4me1 (Abcam, ab8895), rabbit polyclonal H3K4me2 (Abcam, ab7766), rabbit polyclonal H3K4me3 (Abcam ab8580), rabbit polyclonal H3K27ac (Abcam, ab4729), and rabbit polyclonal RAD21 (Abcam, ab992). Antibodies were coated to the beads using protein G magnetic beads for 2 h and then washed three times to remove nonspecific binding.

Immunoprecipitated chromatin fragments were subjected to ChIA-PET library construction following the protocol as previously described (Fullwood et al. 2009). Briefly, the chromatin DNA fragments bound to antibody beads were divided into two aliquots for DNA linker ligation. Biotinylated linkers,
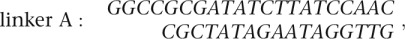

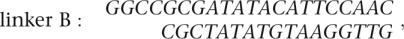
were ligated in 16°C overnight. The two aliquots were combined and subjected to proximity ligation in diluted ligation buffer in 16°C overnight. Crosslinking was reversed using proteinase K. DNA was enzymatically cleaved with MmeI in 37°C for 1 h. DNA fragments with attached linkers were purified using streptavidin beads. Using the resulting fragments, we created sequencing libraries and sequenced them using an Illumina HiSeq 2000.

### Interaction calling

Paired-end reads were processed to remove linker sequences and aligned to the human genome (hg19) using Bowtie (Langmead et al. 2009). Aligned reads were filtered to remove unaligned reads, reads mapping to multiple genomic loci, duplicate reads, and reads that could arise from self circularization. Filtered reads, as well as those resulting from self-circularization, were used to call peaks using either MACS2 or SICER (Zhang et al. 2008; Zang et al. 2009). PETs that did not connect two binding sites (±1500 bp) were removed. The remaining PETs were used to determine interactions. A distance matched rewired (DMR) data set was created to determine interaction *Z*-scores and allow for FDR estimation. Interactions between any two binding sites were scored and filtered such that no more than 10% of called interactions corresponded to the DMR data set. See Supplemental Information for more details.

### Annotation of “regulatory elements”

DHSs for K562 were intersected with “combined” chromatin state calls via integration of both ChromHMM and Segway outputs as determined by Hoffman et al. (2013). DHSs were annotated as the chromatin state that they overlapped the most. DHSs that did not overlap any chromatin state calls were ignored. The remaining annotated DHSs were considered the complete list of regulatory elements in K562 cells.

### Comparison to Hi-C and 5C data sets

We intersected our interactions with TADs determined by Dixon et al. (2012). Boundaries of domains were converted to hg19 using the UCSC liftOver tool. BEDTools intersectBed function (Quinlan and Hall 2010) was used to determine which interactions crossed boundaries by > 20 kb, and these were considered “inter TAD” interactions. In order to determine overlap with TAD boundaries, each boundary region (single base pair) was first padded by 20 kb on either side.

In order to determine overlap with 5C interactions, we intersected our results with those from Sanyal et al. (2012). We first added 10 kb in both directions to each end of our ChIA-PET interactions. We then filtered these interactions to include only those tested by Sanyal et al. (2012). Of the remaining interactions, we determined what percentage of these interactions was also called an interaction by 5C. To determine expected overlap we built random sets of 5C interaction from the sets of 5C interactions tested that had the same distribution of interactions distances as the observed data. One thousand sets of random interactions were generated to assess significance.

### Enrichment of TFs at interacting loci

To test for enrichment of TFs at interacting loci, we first intersected CPBS with TF binding sites as determined by ChIP-seq acquired by the ENCODE Project Consortium (2012). For each TF we asked what percentage of interacting loci overlaps a ChIP-seq peak. To determine expected overlap, we performed the same analysis on a randomly selected set of CPBS. Significance of enrichment was determined by Fisher’s exact test and corrected for multiple hypothesis testing using the Bonferroni method. TFs were annotated as highly enriched if the *P*-value was ≤0.01, the log_2_(observed/expected) was greater than 0.35, and if the TF was present in at least 35% of interacting regions. TFs were annotated as weakly enriched if the *P*-value was ≤0.01 but did not meet the other criteria. If the *P*-value of enrichment was > 0.01 the enrichment was categorized as “none.”

### Self-organizing maps

Self-organizing maps (SOM) were constructed for each of the six ChIA-PET data sets using the R package “kohonen” (Kohonen 2001). The package was modified slightly to allow each neuron to be plotted as a hexagon rather than a circle. For each CPBS we determined which TFs were and were not bound and constructed a matrix of ones and zeros representing this information. We then used the R package “kohonen” to generate 10 SOMs and selected the one with the minimum mean distance metric.

SOMs were made individually for each of the six data sets. Neurons enriched for interactions were determined by Fisher’s exact test with Benjamini-Hochberg correction (*P* < 0.01) and filtered to include only those with fold enrichment greater than two. For generation of the heatmap in Figure 2, enriched neurons from all six SOMs were combined.

### HOT region analysis

To determine the number of observed HOT regions involved in interactions, we intersected our interacting loci with HOT regions determined by the ENCODE Project Consortium (2012). To determine the number of expected HOT regions involved in interactions, we generated a random set of interacting loci from our CPBS and intersected these with HOT regions determined by the ENCODE Project Consortium. The significance of the difference between the two values was determined by Fisher’s exact test.

To determine the number of expected HOT–HOT regions, we generated 10,000 random sets of interactions by randomly rewiring interactions. The mean value was used as the null value of expected interactions linking two HOT regions. These random data sets of interactions were also used to assess significance.

### Effect of distal chromatin state on gene expression

Promoters were defined as the 2000-kb regions upstream of GENCODE V7 genes. For each gene whose promoter was involved in an interaction we determined the chromatin states of the distal regulatory region by intersecting with our annotated DHSs. It was possible for one gene to be associated with multiple distal chromatin states. RPKMs for each gene in K562 were downloaded from the ENCODE website.

### Cell-type-specific gene expression

Gene expression data for 12 cell lines were downloaded from the ENCODE website (http://genome.ucsc.edu/ENCODE/downloads.html). Genes were considered cell K562-specific if they were detected with > 10 RPKM in K562 and ≤ 10 RPKM in all other cell lines. Genes were considered broadly expressed if they were detected at > 10 RPKM in all 12 cell lines. Genes were annotated based upon which types of regulatory elements were present at the distal end of an interaction. Categories are not exclusive as gene promoters can interact with multiple different types of regulatory elements. Correlation between cell-type-specific gene expression and interaction with each distal chromatin state was determined using Fisher’s exact test (*P* < 0.05).

### Gene ontology enrichment analysis of genes with TSS-E interactions

Gene promoters were defined as the 2-kb region upstream of GENCODE V7 genes. Genes whose promoters interacted with at least one enhancer were included. All genes whose promoters were bound by one of the six TFs were used as the background. GO analyses were performed using the R package “topGO” available from Bioconductor using the GOslim annotations. *P*-values were corrected using the Benjamini-Hochberg method. GO terms enriched in sets of genes whose promoters interacted with enhancers were filtered for *P*-values < 0.05 with a fold change > 1.2.

### Clustering interactions by histone marks

Histone ChIP-seq files for 11 histone marks were downloaded from the ENCODE website (http://genome.ucsc.edu/ENCODE/downloads.html) in bam format. Intrachromosomal interactions from all six ChIA-PET data sets were combined. Thirty equally sized genomic bins were generated around each interaction; 10 bins in between the two interacting sites and 10 on either side of the loop. Reads were counted for each histone mark in each bin. Bins were normalized across marks and across interactions as previously described (Handoko et al. 2011). Interactions were clustered into eight clusters using k-means clustering. GO enrichment was done using the R package “topGO” and the GOslim annotations. *P*-values were corrected using the Benjamini-Hochberg method.

### Construction of regulatory networks

Proximal regulatory networks were constructed using TF biding peaks for 102 factors in K562 cells downloaded from the ENCODE website (http://genome.ucsc.edu/ENCODE/downloads.html). Edges were constructed from TFs to genes whose promoters to which they were bound. Promoters were determined by extending 5 kb in both directions from the TSS as defined by GENCODE V7 annotations.

Distal regulatory networks were built by combining TF binding peaks for 102 factors in K562 with our ChIA-PET interactions. Edges were constructed when TFs bound to distal regions that interacted with gene promoters. Interactions that connected two gene promoters were not used in the construction of distal regulatory networks. Combined regulatory networks were built by taking the union of edges from the proximal and distal regulatory networks. In addition to these three “total” networks, we constructed three “TF-only” networks for which we removed all nodes except for the 102 TFs for which we have TF binding data.

### Construction of hierarchical networks

Hierarchical networks of TF binding have been utilized by Gerstein et al. (2012) and others in order to understand the global structure of regulation. We built hierarchical networks using the same approach. Using the TF-only networks, nodes were organized into three tiers using a simulated annealing algorithm that maximized downward-pointing edges. Each network was built five times. The network with the most downward-pointing edges was used for further analysis.

### GO enrichment comparisons of proximal vs. distal regulatory networks

GO enrichments for proximal and distal targets of each TF were determined using R package “topGO” and the GOslim annotations. *P*-values were corrected using the Benjamini-Hochberg method.

To compare proximal and distal GO enrichments we first filtered TFs for only those with outward edges in both networks. We calculated *P*-values of GO enrichments as described above. The *P*-value ratio was calculated as the log_2_ ratio of proximal vs. distal *P*-values.

## Data access

The ChIA-PET data from this study have been submitted to the NCBI Gene Expression Omnibus (GEO; http://www.ncbi.nlm.nih.gov/geo/) under accession number GSE59395 and to the ENCODE Data Coordination Center (https://www.encodeproject.org/datasets/) under accession number ENCSR727WCB.

## Competing interest statement

M.P.S. is a cofounder and scientific advisory board (SAB) member of Personalis. He is also on the SAB of Genapsys.

## Supplementary Material

Supplemental Material
