# Supplementary material for: Genome-wide map of regulatory interactions in the human genome
Source: Genome Res. 2014 Dec;24(12):1905–17. doi: 10.1101/gr.176586.114 (PMC4248309; doi:10.1101/gr.176586.114)
Supplement: Supplemental Material [file supp_24_12_1905__index.html]

Supplemental Material 

# Genome-wide map of regulatory interactions in the human genome

## Supplemental Material

**Files in this Data Supplement:**

- Supplemental Figure1.eps
- Supplemental Figure2.eps
- Supplemental Figure3.eps
- Supplemental Figure4.eps
- Supplemental Figure5.eps
- Supplemental Information.docx
- TableS1.xlsx
