# Supplementary material for: Genome-wide map of regulatory interactions in the human genome
Source: Genome Res. 2014 Dec;24(12):1905–17. doi: 10.1101/gr.176586.114 (PMC4248309; doi:10.1101/gr.176586.114)
Supplement: Supplemental Material [file supp_gr.176586.114_Supplemental_Information.docx]

**EXTENDED EXPERIMENTAL PROCEDURES**

**Interaction Calling**

Interactions were determined in five distinct steps.

*PET parsing.*  Fastq files were parsed into three files depending on whether they contained the same pair of linkers (AA or BB), chimeric pairs of linkers (AB or BA), or if they were ambiguous (XX, AX, XA, BX, XB).  Matches to linkers sequence required a perfect match to fist 10 positions in the linker (GTTGGATAAG or GTTGGAATGT). PETs were trimmed to remove linker sequences and PETs containing trimmed reads shorter than 15 bp at either end were removed. Only PETs containing AA or BB were used for further analyses.

*Read Alignment.* Reads were aligned using Bowtie (version 0.12.7) allowing zero mismatches (arguments: -v 2 -S -k 1--best --strata -m 1)([Langmead et al. 2009](#_ENREF_10)).

*PET filtering*. Aligned PETs were filtered to remove PETs that contained reads that were unmapped or that mapped to multiple locations in the genome.  We conservatively removed PETs that represent duplicate reads by filtering any PETs whose reads start within +/- 2 bases of another PET and who have the same pair of linkers.  PETs comprised of reads that map within a certain genomic distance are likely to arise from self-circularization rather than ligation to an interacting fragment.  In order to avoid false positives introduced by this artifact we filtered out short distance interactions.  To accurately determine the cutoff for these interactions we exploited a read orientation bias of self-circularization reads.  PETs arising from self-circularization always contain reads that map to opposite strands (+- or -+).  PETs arising from ligation of interacting fragments should exhibit no preference for the same or opposite strands.  We determine a minimum PET cutoff above which we see no observable bias for opposite strand PETs.  To determine the expected ratio of opposite to same orientation reads we calculate the mean and standard deviation of the log2 ratio of same to opposite strand PETs in all bins greater than ~400 kb.  This value is typically very close to zero.  Our minimum PET distance is set as the size corresponding to the smallest bin that falls within 2 standard deviations of the expected ratio.  PETs with distance below this distance are removed.

*Peak Calling.* In order to determine binding sites we use MACS2 (version 2.0) for RAD21 and POLR2A and SICER (version 1.1) for histone marks ([Zhang et al. 2008](#_ENREF_17); [Zang et al. 2009](#_ENREF_16)).  P-value cutoffs for MACS2 and SICER were 0.0001 and 0.01 respectively.  The binding sites determined from this analysis are heretofore referred to as ‘ChIA-PET binding sites’ or CPBS.

*Interaction Calling.* Peaks were extended in both directions by 1500.  PETs that overlapped peaks at both ends were determined and those that did not were removed.  Raw interaction scores were determined by the following formula:

$$S=\frac{L^{2}}{(P_{1} \times P_{2})}$$

where S is raw interaction score, L is number of PETs linking the two peaks, and P_1_ and P_2_ are number of PETs overlapping each of the two regions in question.

Previous studies have shown that interactions between genomic loci show a strong dependence on linear genomic distance([Dekker et al. 2002](#_ENREF_3); [Sanyal et al. 2012](#_ENREF_14)).

To account for this we developed a resampling method to construct a distance-matched rewired (DMR) ChIA-PET data set that allows for distance-corrected scoring of interaction frequencies as well as estimation of false discovery rate (FDR). Rewired ChIA-PET data sets are constructed in a two-step process.  First, paired-end reads are disassociated from each other and re-paired with replacement generating a large set of rewired PETs (200 times the original size of the data set).  Second, the distribution of PET distances from the original data set is determined.  Distances are drawn at random from this distribution and matched to the rewired PET that has the most similar distance.  This rewired PET gets included in our final set of DMR PETs. This procedure is repeated until the number of DMR PETs is equal to the number of observed PETs. Interaction frequencies and raw scores are calculated for both observed and DMR data sets as described above. Z-scores are calculated for both observed and DMR data sets by comparison to the weighted mean and standard deviation of the DMR data set and a Z-score cutoff is set so that a user defined fraction of the interactions with a Z-score greater than that cutoff come from the rewired data set.  This allows for data sets to be filtered to a user-defined FDR.  In order to more easily compare with recent 5C data sets, that had FDRs of 18% and 9%, we chose to filter our data sets to an FDR of 10%.

For better discrimination of real vs random interactions we iteratively applied minimum PET cutoffs (minimum values of L) and maximum distance cutoffs.  For each combination of cutoffs FDR filtering was applied.  The combination of cutoffs that generated the maximum number interactions at a fixed FDR (10%) was used.

Though this method can detect interchromosomal interactions very few were determined (287) and they had significantly lower Z-scores than intrachromosomal interactions and are more likely to be false positives (p < 2 x 10^-16^, Wilcoxon signed-rank test). Therefore, we excluded interchromosomal interactions from all of our analyses.

Sequencing replicates were combined at the fastq level.  Biological replicates were combined after PET filtering.  And data from different factors were combined after all steps of interaction calling were completed

**Detection of differential interactions between K562 and GM12878 cells**

- 1. **Determining pairs of loci for differential analysis**

In order to fairly compare the two data sets we first merged all reads from both the K562 and GM12878 RAD21 ChIA-PET experiments and determined a set of putative interacting loci. To determine which pairs of loci to compare we then used a clustering method previously described ([Ester M 1996](#_ENREF_5)). Each PET is represented as a point in the two-dimensional Euclidean space and clustered by DBSCAN (Density-Based Spatial Clustering of Applications with Noise) described in ([Ester M 1996](#_ENREF_5)) with the python package Scikit-learn ([Pedregosa 2011](#_ENREF_13)). The DBSCAN parameters are set as: the size of the ε-neighborhood of a point, Eps = 1500 bp; and the minimum number of points required to form a cluster, MinPts = 3 ([Chepelev et al. 2012](#_ENREF_1)). Those PETs that do not belong to any PET clusters were removed since they are likely random ligation products formed in solution. These PET clusters were then tested for differential interaction between the two cell types.

**1.2 Modeling variability as a function of PET counts**

In order to distinguish true quantitative differences from technical variability we first needed to model variability as a function of PET counts. We reasoned that pairs of loci linked by higher PET counts should be les variable and less subject to technical variability than those linked by lower PET counts. To accurately model this distribution we generated MA plots using the following equations.

For a specific PET cluster (A, B), denote

$c_{\mathrm{AB}}^{(i)}, i=1,2$: PET counts between region A and B for experiment i (i=1,2 refers to either K562 or GM12878, respectively)

$c_{A}^{(i)}, i=1,2$: PET end counts within region A for experiment i

$c_{B}^{(i)}, i=1,2$: PET end counts within region B for experiment i

$p_{\mathrm{AB}}^{(i)}, i=1,2$: Interacting probability between region A and B for experiment i

$n_{i}, i=1,2$: Total PET counts that belong to PET clusters for experiment i

We assume $c_{\mathrm{AB}}^{(i)}\sim Binom\left( n_{i}{,p}_{\mathrm{AB}}^{\left( i \right)} \right), i=1,2$

By Central Limit Theorem, when $n_{i}$ is large enough:

$$\sqrt{n_{i}}\left( \frac{c_{\mathrm{AB}}^{\left( i \right)}}{n_{i}}-p_{\mathrm{AB}}^{\left( i \right)} \right)\sim Norm\left( 0,p_{\mathrm{AB}}^{\left( i \right)}\left( 1-p_{\mathrm{AB}}^{\left( i \right)} \right) \right), i=1,2$$

Using function $g_{i}\left( x \right)=log(n_{i}x)$, and according to Delta Method.

$$\sqrt{n_{i}}\left( \log\left( n_{i}\frac{c_{\mathrm{AB}}^{\left( i \right)}}{n_{i}} \right)-{\log(n_{i}p}_{\mathrm{AB}}^{\left( i \right)}) \right)$$

$$=\sqrt{n_{i}}\left( g_{i}\left( \frac{c_{\mathrm{AB}}^{\left( i \right)}}{n_{i}} \right)-{g_{i}(p}_{\mathrm{AB}}^{\left( i \right)}) \right)$$

$$\sim Norm\left( 0, p_{\mathrm{AB}}^{\left( i \right)}\left( 1-p_{\mathrm{AB}}^{\left( i \right)} \right)\left[ g_{i}^{'}\left( p_{\mathrm{AB}}^{\left( i \right)} \right) \right]^{2} \right)$$

$$=Norm\left( 0,\frac{1-p_{\mathrm{AB}}^{\left( i \right)}}{p_{\mathrm{AB}}^{\left( i \right)}} \right), i=1,2$$

Let $X=\log\left( c_{\mathrm{AB}}^{\left( 1 \right)} \right), Y=\log\left( c_{\mathrm{AB}}^{\left( 2 \right)} \right)$, then

$$X\sim Norm\left( \mu_{X}=\log\left( {n_{1}p}_{\mathrm{AB}}^{\left( 1 \right)} \right), \sigma_{X}^{2}=\frac{1-p_{\mathrm{AB}}^{\left( 1 \right)}}{n_{1}p_{\mathrm{AB}}^{\left( 1 \right)}} \right)$$

$$Y\sim Norm\left( \mu_{Y}=\log\left( {n_{2}p}_{\mathrm{AB}}^{\left( 2 \right)} \right), \sigma_{Y}^{2}=\frac{1-p_{\mathrm{AB}}^{\left( 2 \right)}}{n_{2}p_{\mathrm{AB}}^{\left( 2 \right)}} \right)$$

When $n_{i},n_{2}$ are large enough. Assume $c_{\mathrm{AB}}^{\left( 1 \right)}$ and $c_{\mathrm{AB}}^{\left( 2 \right)}$ are independent and denote $M=X-Y$, $A=(X+Y)/2$, then

$$M\sim Norm({\mu_{M}=\mu}_{X}-\mu_{Y},\sigma_{M}^{2}{=\sigma}_{X}^{2}+\sigma_{Y}^{2})$$

$$A\sim Norm\left( \mu_{A}=\frac{\mu_{X}+\mu_{Y}}{2},\sigma_{A}^{2}=\frac{\sigma_{X}^{2}+\sigma_{Y}^{2}}{4} \right)$$

After some derivation ([Wang et al. 2010](#_ENREF_15)), the conditional distribution of $M|A$ follows

$$M|(A=a)\sim Norm\left( \mu_{X}-\mu_{Y}+2\frac{\sigma_{X}^{2}-\sigma_{Y}^{2}}{\sigma_{X}^{2}+\sigma_{Y}^{2}}\left( a-\frac{\mu_{X}+\mu_{Y}}{2} \right), \frac{4\sigma_{X}^{2}\sigma_{Y}^{2}}{\sigma_{X}^{2}+\sigma_{Y}^{2}} \right)$$

**1.3 Assigning statistical confidence estimates to differential interactions**

Using the estimates of variability determined by our model we can calculate Z scores and ultimately p values to describe the statistical confidence of each differential interaction. However, differential binding events can lead to differences in contact frequencies and must be accounted for. In order to account for differential binding we first model the correlation between peak depths (C_A_ and C_B_) and contact frequencies (p_AB_).

Assume $p_{\mathrm{AB}}^{\left( i \right)}=p_{0AB}^{\left( i \right)}f\left( c_{A}^{(i)},c_{B}^{(i)} \right), i=1,2$, in which $p_{0AB}^{\left( i \right)}$ is the interacting probability between region A and B after removing the factor of protein binding intensity $f\left( c_{A},c_{B} \right)$. Then the hypothesis to test whether (A, B) is a differential interaction between two cells is ${H_{0}: p}_{0AB}^{\left( 1 \right)}=p_{0AB}^{\left( 2 \right)}=p_{0}$ versus ${H_{1}: p}_{0AB}^{\left( 1 \right)}\neq p_{0AB}^{\left( 2 \right)}$.

Based on the above deduction,

$${2\mu}_{A}=\mu_{X}+\mu_{Y}=\log\left( n_{1}p_{\mathrm{AB}}^{\left( 1 \right)}n_{2}p_{\mathrm{AB}}^{\left( 2 \right)} \right)=\log\left( n_{1}n_{2}f\left( c_{A}^{\left( 1 \right)},c_{B}^{\left( 2 \right)} \right)f\left( c_{A}^{\left( 2 \right)},c_{B}^{\left( 2 \right)} \right)p_{0}^{2} \right)$$

Thus

$$p_{0}=\sqrt{\frac{\exp(2\mu_{A})}{n_{1}n_{2}f\left( c_{A}^{\left( 1 \right)},c_{B}^{\left( 2 \right)} \right)f\left( c_{A}^{\left( 2 \right)},c_{B}^{\left( 2 \right)} \right)}}$$

Denote $R=\log\left( \frac{{c_{A}^{\left( 1 \right)}c}_{B}^{\left( 1 \right)}}{{c_{A}^{\left( 2 \right)}c}_{B}^{\left( 2 \right)}} \right)$ and assume $\log\left( \frac{{{f(c}_{A}^{\left( 1 \right)}c}_{B}^{\left( 1 \right)})}{f({c_{A}^{\left( 2 \right)}c}_{B}^{\left( 2 \right)})} \right)=h\left( \log\left( \frac{{c_{A}^{\left( 1 \right)}c}_{B}^{\left( 1 \right)}}{{c_{A}^{\left( 2 \right)}c}_{B}^{\left( 2 \right)}} \right) \right)=h(R)$.

Use $a$ as an estimate of $\mu_{A}$, then

$$\hat{E}(M│A=a)=\log\left( \frac{n_{1}}{n_{2}} \right)+h\left( R \right)$$

$$\hat{D}\left( M | A=a \right)=\frac{4\left( \exp\left( \frac{h\left( R \right)}{2} \right)-\alpha\right)\left( 1-\alpha\exp\left( \frac{h\left( R \right)}{2} \right) \right)}{\alpha\left( n_{1}\exp\left( h\left( R \right) \right)+n_{2}-\alpha\left( n_{1}+n_{2} \right)\exp\left( \frac{h\left( R \right)}{2} \right) \right)}, \alpha=\sqrt{\frac{\exp(2a)}{n_{1}n_{2}}}$$

$h\left( R \right)$ is a logistic-like function, thus we set

$$h\left( R \right)=\beta_{2}\frac{1-\exp(\beta_{0}R+\beta_{1})}{1+exp(\beta_{0}R+\beta_{1})}$$

Parameters of $\beta_{0},\beta_{1},\beta_{2}$ can be determined by maximizing the log-likelihood function

$$l\left( \beta_{0},\beta_{1},\beta_{2} | m_{k}, a_{k} \right)=\sum_{k} \log\left( P\left( m_{k} | a_{k} \right) \right)$$

Then by using the two estimations of expectation and variation, we can calculate the Z-score and convert it to the two-side p-value to describe whether PET cluster (A, B) is a differential interaction between K562 and GM12878 cells or not.

$$Z=\left| \frac{m-\hat{E}\left( M | A=a \right)}{\sqrt{\hat{D}\left( M | A=a \right)}} \right|$$

- 1. **FDR estimation**

Finally, we estimate the False Discovery Rate (FDR) of differential interactions by comparing observed results to results from a permutated data set. We randomly shuffle the label of PETs that belong to K562 or GM12878 experiment, and calculate the permutated Z-score ($Z_{p}$) again. Then FDR is estimated by comparing the observed Z-score ($Z_{o}$) with permutated Z-score.

$$\mathrm{FDR}\left( z \right)=\frac{\#\{Z_{p}>z\}}{\#\{Z_{o}>z\}}$$

In which $\#\{\cdot\}$ is a counting function. We filter observed differential interactions to an FDR of 0.05.

**SUPPLEMENTAL TABLES**

| **Cells** | **Factor** | **Bio Rep** | **Tech Rep** | **Sequencing Depth** | **Same PETs** | **Chimeric PETs** | **Ambiguous PETs** | **Non-uniquely mapped PETs** | **Un-mapped PETs** | **Duplicate PETs** | **Self-ligated PETs** | **Intra-chromosomal PETs** | **Inter-chromosomal PETs** | **Peaks** | **Interactions** |
| --- | --- | --- | --- | --- | --- | --- | --- | --- | --- | --- | --- | --- | --- | --- | --- |
| K562 | **H3K27ac** | 1 | 1 | 165109173 | 79722577 | 54750055 | 30636541 | 54489345 | 17864 | 948089 | 3487570 | 1411505 | 19368204 | 74011 | 579 |
| K562 | **H3K27ac** | 2 | 1 | 177322797 | 131390006 | 27235810 | 18696981 | 87947223 | 40433 | 21345695 | 1583530 | 2923249 | 17549876 | 48193 | 993 |
| K562 | **H3K4me1** | 1 | 1 | 162190720 | 111183880 | 31486229 | 19520611 | 72640135 | 30237 | 823103 | 3148349 | 2230690 | 32311366 | 74039 | 492 |
| K562 | **H3K4me1** | 2 | 1 | 148997533 | 112686495 | 19821264 | 16489774 | 75649209 | 32946 | 4196124 | 7715087 | 2290646 | 22802483 | 67763 | 3137 |
| K562 | **H3K4me2** | 1 | 1 | 133923924 | 104263094 | 14050408 | 15610422 | 66495132 | 28978 | 15452476 | 1470806 | 1689123 | 19126579 | 51455 | 287 |
| K562 | **H3K4me2** | 2 | 1 | 168742629 | 127227629 | 18238500 | 23276500 | 81903555 | 148326 | 20662035 | 2014471 | 1442184 | 21057058 | 49666 | 379 |
| K562 | **H3K4me3** | 1 | 1 | 150470693 | 97438798 | 36903231 | 16128664 | 60294912 | 12862 | 25719458 | 3656146 | 988794 | 6766626 | 19285 | 1179 |
| K562 | **H3K4me3** | 2 | 1 | 164584679 | 104878365 | 31507991 | 28198323 | 72769843 | 26592 | 26255888 | 872069 | 462849 | 4491124 | 16845 | 190 |
| K562 | **Pol2A** | 1 | 1 | 180430214 | 150231459 | 6149148 | 24049607 | 97669589 | 62791 | 29383443 | 7745028 | 1620423 | 13750185 | 28906 | 2782 |
| K562 | **Pol2A** | 2 | 1 | 72576997 | 62118832 | 2570540 | 7887625 | 40407462 | 22731 | 8607792 | 4079368 | 923646 | 8077833 | 21567 | 1187 |
| K562 | **Pol2A** | 2 | 2 | 182627883 | 154157458 | 7334726 | 21135699 | 100367933 | 39942 | 34586770 | 5940180 | 1331197 | 11891436 | 26140 | 2211 |
| K562 | **RAD21** | 1 | 1 | 115140018 | 86819909 | 1099957 | 27220152 | 59005051 | 71532 | 21379381 | 1203125 | 656550 | 4504270 | 22899 | 3315 |
| K562 | **RAD21** | 1 | 2 | 138473050 | 89125493 | 1137119 | 48210438 | 60514193 | 13103 | 22217994 | 1201248 | 667904 | 4511051 | 22982 | 3632 |
| K562 | **RAD21** | 2 | 1 | 148398674 | 121245356 | 7572488 | 19580830 | 78168531 | 61271 | 34484473 | 3088468 | 1293736 | 4148877 | 42454 | 11134 |
| GM12878 | RAD21 | 1 | 1 | 225434403 | 154049990 | 46248174 | 25136239 | 101742999 | 110014 | 30392738 | 3925495 | 1887906 | 15990838 | 58316 | 11364 |
| GM12878 | RAD21 | 2 | 1 | 138472402 | 111086757 | 4375566 | 23010079 | 76646643 | 19760 | 20697497 | 2829649 | 1384418 | 9508790 | 30721 | 8689 |

**Table S2. ChIA-PET statistics for individual data sets.**

| **Factor** | **Binding peaks** | **Interactions** | **General Interactions** | **Factor-Specific Interactions** |
| --- | --- | --- | --- | --- |
| **H3K4me2** | 51095 | 513 | 276 | 237 |
| **H3K4me3** | 22463 | 1360 | 764 | 596 |
| **H3K27ac** | 69138 | 2231 | 1344 | 887 |
| **H3K4me1** | 86567 | 5012 | 3235 | 1777 |
| **POLR2A** | 40581 | 5549 | 3376 | 2173 |
| **RAD21** | 48894 | 14701 | 10012 | 4689 |

**Table S3. ChIA-PET statistics for individual factors.**

|  |  | **% of each element overlapped by a peak** | | | | | | | **% of each element overlapped by an anchor region** | | | | | | |
| --- | --- | --- | --- | --- | --- | --- | --- | --- | --- | --- | --- | --- | --- | --- | --- |
|  | **Total** | **H3K27ac** | **H3K4me1** | **H3K4me2** | **H3K4me3** | **Pol2A** | **RAD21** | **all peaks** | **H3K27ac** | **H3K4me1** | **H3K4me2** | **H3K4me3** | **POLR2A** | **RAD21** | **all anchors** |
| **CTCF** | 29389 | 98.7 | 96.6 | 99.4 | 95.3 | 83.6 | 28.2 | 99.7 | 5.3 | 9.5 | 2.1 | 12.4 | 20.2 | 8.7 | 35.5 |
| **E** | 35176 | 93.3 | 96.1 | 92.6 | 36.1 | 41.7 | 14.5 | 98 | 5.9 | 8.1 | 2.2 | 5.2 | 6.5 | 3.6 | 21 |
| **PF** | 1079 | 68.8 | 85.1 | 54.5 | 8 | 14.7 | 4.5 | 86.2 | 4.1 | 6.8 | 0.9 | 1 | 1.5 | 0.8 | 11.9 |
| **R** | 22072 | 39.3 | 50.2 | 28.8 | 8.6 | 25.8 | 76.2 | 84.7 | 6.5 | 13.8 | 1.5 | 1.9 | 9.6 | 37.4 | 43.9 |
| **T** | 12082 | 62 | 70.4 | 71.4 | 35.7 | 14.6 | 0.8 | 79.3 | 2.3 | 5.8 | 0.9 | 3.3 | 0.6 | 0.1 | 9.7 |
| **TSS** | 42999 | 48.3 | 59.4 | 27.3 | 10.9 | 7.3 | 1 | 64 | 2.8 | 5.3 | 0.7 | 1.4 | 0.9 | 0.3 | 8.7 |
| **WE** | 27074 | 15.9 | 25.7 | 12.8 | 3.6 | 1.2 | 0.7 | 28.9 | 1 | 2.4 | 0.2 | 0.3 | 0.1 | 0.2 | 3.7 |
| **Total** | 169871 | 68 | 74.6 | 62.1 | 35.8 | 37.4 | 24.2 | 82.7 | 4.7 | 8.3 | 1.5 | 4.9 | 8.5 | 9.6 | 24 |

**Table S4. Comparison of ChIA-PET peaks and interactions with various genomic elements.**

**SUPPLEMENTAL FIGURE CAPTIONS**

**Figure S1. Comparison of ChIA-PET and 5C data sets.**

(A) Percent of ChIA-PET interactions also found by 5C.  Only ChIA-PET interactions that were tested by 5C were considered.  Grey bars represent expected percentages generated by randomly selecting interactions from tested 5C region while retaining the same distribution of interaction distances.  Stars represent a p-value < 0.05 (permutation testing, 1000 permutations).  Black bars represent ChIA-PET data published by Li et al. (B) Density plot depicting the distribution of interacting distances. (C) Density plot depicting the distribution of anchor region sizes. (D) Barplot depicting the number of general interactions (those found in more than one data set) and factor specific interactions (those found only in one data set). (E) Plot depicting the overlap between two biological replicates as a function of the highest N% of interactions.

**Figure S2. TF enrichment at interacting loci.**

(A) TF enrichment at interacting loci for each individual data set.  X-axis represented the log2 ratio of observed vs expected TFs binding peaks overlapping interacting loci.  Y-axis represents the number of interacting regions at which factor is bound.  Colors of circles represent the level of enrichment (see Supplementary Methods).

(B) Box and whisker plot of Z-scores of interactions that overlap a RAD21 peak at both, one, or neither end of an interaction for each individual data set. Asterisks mark significant differences (p < 0.05, Wilcoxon signed-rank test).

**Figure S3. Characteristics of hierarchical networks.**

(A) Based on the GM12878 networks, the percentage of targets found in the distal, proximal, or both networks are depicted for each TF.

(B) Based on the GM12878 networks, hierarchical networks built from proximal, distal, and combined TF only networks are shown.  Blue lines represent downward edges, red lines represent upward edges, and grey lines represent lateral edges.  The colors of the nodes represent the tier that the node resides in in the proximal network.  The size of the node represents the degree (total number of inward and outward edges) for each node in that network.

(C) Based on the GM12878 networks, box and whisker plots depicting the degree (total inward and outward edges) of nodes in each tier of each hierarchical network.

(D) Plot depicting the number of TFs in the top, middle, and bottom tier of each hierarchical network determined from K562 cells. (E) Plot depicting the number of TFs in the top, middle, and bottom tier of each hierarchical network determined from GM12878 cells.

**Figure S4. Proximal vs distal regulation of GO terms.**

(A) Six plots highlighting examples of GO terms that exhibit different profiles of enrichment.  Each circle represents a TF.  The size of the circle represents the number of targets of each TF corresponding to that GO term.  The color of the circle represents the relative enrichment using the same scale as shown in panel A.

(B) Six plots highlighting examples of TFs that exhibit different profiles of GO term enrichment.  Each circle represents a GO term.  The size of the circle represents the number of targets in that GO term that that TF factor regulates.  The color of the circle represents the relative enrichment (log2(direct p−value / indirect p−value)).

**Figure S5. Proximal vs distal regulation of GO terms in GM12878 cells.**

(A) Heatmap comparing enrichment of GO terms in proximal vs distal targets of each TF.  Each row corresponds to a GO term.  Each column corresponds to a transcription factor.  Red indicates greater enrichment in distal targets.  Blue represents greater enrichment in proximal targets.

(B) Three plots highlighting examples of GO terms that exhibit different profiles of enrichment.  Each circle represents a TF.  The size of the circle represents the number of targets in that GO term that TF factor regulates (both proximally and distally).  The color of the circle represents the relative enrichment (proximal vs distal) using the same scale as shown in panel A.

**SUPPLEMENTAL REFERENCES**

Chepelev I, Wei G, Wangsa D, Tang Q, Zhao K. 2012. Characterization of genome-wide enhancer-promoter interactions reveals co-expression of interacting genes and modes of higher order chromatin organization. *Cell research* **22**(3): 490-503.

Consortium EP, Bernstein BE, Birney E, Dunham I, Green ED, Gunter C, Snyder M. 2012. An integrated encyclopedia of DNA elements in the human genome. *Nature* **489**(7414): 57-74.

Dekker J, Rippe K, Dekker M, Kleckner N. 2002. Capturing chromosome conformation. *Science* **295**(5558): 1306-1311.

Dixon JR, Selvaraj S, Yue F, Kim A, Li Y, Shen Y, Hu M, Liu JS, Ren B. 2012. Topological domains in mammalian genomes identified by analysis of chromatin interactions. *Nature* **485**(7398): 376-380.

Ester M KH, Sander J, Xu X. 1996. A density-based algorithm for discovering cluster in large saptial databased with noise. *Proceeding of 2nd international conference on knowledge discovery and data mining*: 226-231.

Gerstein MB, Kundaje A, Hariharan M, Landt SG, Yan KK, Cheng C, Mu XJ, Khurana E, Rozowsky J, Alexander R et al. 2012. Architecture of the human regulatory network derived from ENCODE data. *Nature* **489**(7414): 91-100.

Handoko L, Xu H, Li G, Ngan CY, Chew E, Schnapp M, Lee CW, Ye C, Ping JL, Mulawadi F et al. 2011. CTCF-mediated functional chromatin interactome in pluripotent cells. *Nature genetics* **43**(7): 630-638.

Hoffman MM, Ernst J, Wilder SP, Kundaje A, Harris RS, Libbrecht M, Giardine B, Ellenbogen PM, Bilmes JA, Birney E et al. 2013. Integrative annotation of chromatin elements from ENCODE data. *Nucleic acids research* **41**(2): 827-841.

Kohonen T. 2001. *Self-Organizing Maps*. Springer, Berlin.

Langmead B, Trapnell C, Pop M, Salzberg SL. 2009. Ultrafast and memory-efficient alignment of short DNA sequences to the human genome. *Genome biology* **10**(3): R25.

Milo R, Shen-Orr S, Itzkovitz S, Kashtan N, Chklovskii D, Alon U. 2002. Network motifs: simple building blocks of complex networks. *Science* **298**(5594): 824-827.

Neph S, Stergachis AB, Reynolds A, Sandstrom R, Borenstein E, Stamatoyannopoulos JA. 2012. Circuitry and dynamics of human transcription factor regulatory networks. *Cell* **150**(6): 1274-1286.

Pedregosa F. 2011. Scikit-learn:Machin learning in Python. *The journal of Machin Learning Research* **12**: 2825-2830.

Sanyal A, Lajoie BR, Jain G, Dekker J. 2012. The long-range interaction landscape of gene promoters. *Nature* **489**(7414): 109-113.

Wang L, Feng Z, Wang X, Wang X, Zhang X. 2010. DEGseq: an R package for identifying differentially expressed genes from RNA-seq data. *Bioinformatics* **26**(1): 136-138.

Zang C, Schones DE, Zeng C, Cui K, Zhao K, Peng W. 2009. A clustering approach for identification of enriched domains from histone modification ChIP-Seq data. *Bioinformatics* **25**(15): 1952-1958.

Zhang Y, Liu T, Meyer CA, Eeckhoute J, Johnson DS, Bernstein BE, Nusbaum C, Myers RM, Brown M, Li W et al. 2008. Model-based analysis of ChIP-Seq (MACS). *Genome biology* **9**(9): R137.
